# An Automated High-throughput Array Microscope for Cancer Cell Mechanics

**DOI:** 10.1038/srep27371

**Published:** 2016-06-06

**Authors:** Jeremy A. Cribb, Lukas D. Osborne, Kellie Beicker, Matthew Psioda, Jian Chen, E. Timothy O’Brien, Russell M. Taylor II, Leandra Vicci, Joe Ping-Lin Hsiao, Chong Shao, Michael Falvo, Joseph G. Ibrahim, Kris C. Wood, Gerard C. Blobe, Richard Superfine

**Affiliations:** 1 Department of Physics and Astronomy, UNC-Chapel Hill, Chapel Hill, NC, United States of America; 2 Department of Biostatistics, UNC-Chapel Hill, Chapel Hill, NC United States of America; 3 Department of Medicine and Pharmacology and Cancer Biology, Duke University Medical Center, Durham, North Carolina, United States of America; 4 Department of Computer Science, UNC-Chapel Hill, Chapel Hill, NC, United States of America; 5 Department of Biostatistics, The Lineberger Comprehensive Cancer Center, The University of North Carolina School of Medicine, Chapel Hill, North Carolina, United States of America; 6 Department of Pharmacology and Cancer Biology, Duke University, 450 Research Drive, Durham, NC 27710, United States of America

## Abstract

Changes in cellular mechanical properties correlate with the progression of metastatic cancer along the epithelial-to-mesenchymal transition (EMT). Few high-throughput methodologies exist that measure cell compliance, which can be used to understand the impact of genetic alterations or to screen the efficacy of chemotherapeutic agents. We have developed a novel array high-throughput microscope (AHTM) system that combines the convenience of the standard 96-well plate with the ability to image cultured cells and membrane-bound microbeads in twelve independently-focusing channels simultaneously, visiting all wells in eight steps. We use the AHTM and passive bead rheology techniques to determine the relative compliance of human pancreatic ductal epithelial (HPDE) cells, h-TERT transformed HPDE cells (HPNE), and four gain-of-function constructs related to EMT. The AHTM found HPNE, H-ras, Myr-AKT, and Bcl2 transfected cells more compliant relative to controls, consistent with parallel tests using atomic force microscopy and invasion assays, proving the AHTM capable of screening for changes in mechanical phenotype.

Steady increases in the availability and use of high-throughput (HT) systems has accelerated progress in pursuits as diverse as mapping the human genome, studying cancer chemotherapeutic drug-resistance at the individual cellular proteomics level, and identifying pathogenic bacteria in the hospital setting^1^,^2^,^3^. HT systems have also achieved substantial progress in the identification of genetic or biochemical correlates of tumor progression and chemotherapeutic responses in individual cancer cells^4^,^5^. We are interested in developing new methods to characterize the mechanical phenotype of cancer cells, and also in relation to their ability to invade extracellular matrix as part of the epithelial-to-mesenchymal transition (EMT) thought to facilitate metastasis^6^,^7^. Decreases in cell stiffness, or increased compliance, have been correlated with increased invasiveness in many systems, primarily by atomic force microscopy (AFM), magnetic twisting cytometry and magnetic tweezers^6^,^8^,^9^,^10^,^11^,^12^,^13^,^14^. These techniques, while providing excellent measures of compliance, are low-throughput and thus time-intensive. Recent advances in HT methods provide rapid assessment of individual cell stiffness using optical compression^15^ and hydrodynamic stretching^16^,^17^ of cancer cells in suspension, which offer benefits in terms of scalability and speed. However, there are advantages to characterizing cells while they are attached to a substrate, where the cytoskeleton is intact and active signaling occurs through cell-cell and cell-matrix interactions. Such conditions better emulate the tumor microenvironment *in vivo* and allow for studying cellular mechanical properties as a function of substrate chemical and mechanical properties.

Here we introduce an array high-throughput microscope (AHTM) that can assess the stiffness of cancer cells grown in monolayer culture. It combines twelve independently-focusing objectives, two-color epi-fluorescence, high frame rate imaging, automated analysis and lossless data compression. While the AHTM can directly image fluorescently-labeled cells, here we demonstrate its utility for imaging and tracking fluorescent, volume-labeled microbeads attached to cells grown in standard multiwell plates. The motion of attached or internal beads over time serves as a useful indicator of cell compliance vis a vis passive microbead rheology (PBR) techniques^18^,^19^,^20^,^21^. In earlier work, we have shown that increased compliance positively correlates with invasiveness and metastatic potential^20^. Recently, we reported the utility of an earlier version of the AHTM for biofluid rheology^22^. That system did not require each objective to focus independently, since sampling at any height in the fluid was of interest. Cellular rheology, in contrast, demands accurate placement of the plane-of-focus (POF) at the cell surface for each field-of-view (FOV). Our solution incorporates a voltage-controlled liquid lens within each objective to allow independent and automated focus for each FOV. Here we describe the overall structure of the AHTM, its control and image acquisition systems, image calibration, and our data analysis and compression algorithms. We then describe testing the utility of the AHTM in identifying subtle changes in cell compliance produced by the expression of gain-of-function (GOF) versions of single genes involved in cancer biology. We used cultures of normal human pancreatic ductal epithelial (HPDE) cells and the same cells stably infected with genes for activated H-Ras (HRAS^G12V^)^23^, activated Akt (Myr-AKT, myristolated-AKT)^24^,^25^,^26^, Bcl2^27^,^28^,^29^, and activated TßRI (TßRI-T204D)^30^,^31^,^32^,^33^. In each case, the GOF was functionally validated in the stable cell lines using standard methods, the results of which are shown as Supplementary Figs 1 and 2. We compared those changes with an h-TERT immortalized, nestin and K-Ras expressing pancreatic ductal epithelial line (HPNE)^34^. Finally, we assessed each cell derivative “type” for invasiveness using standard low-throughput assays and for stiffness using AFM. We found that the AHTM could consistently distinguish both the relatively subtle changes engendered by single genetic changes, and the larger difference between normal and oncogene-expressing cells.

## Results

### AHTM System

**Figure 1:**
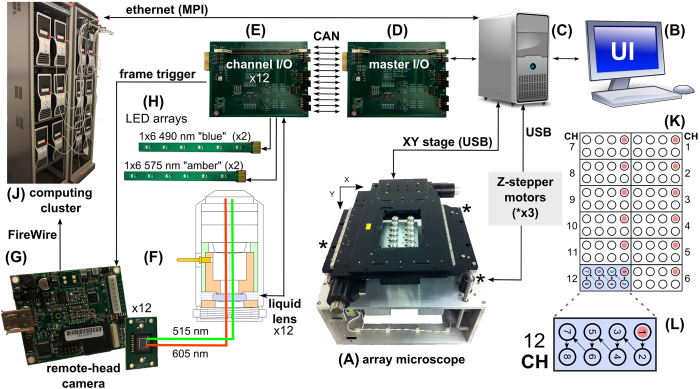
AHTM System Diagram. (**A**) The main body of the AHTM showing the 2 × 6 array of 40× objectives and signal paths between components. A user interface (UI) for the system (**B**) is typically coupled to the master computer (**C**) which controls XY and Z stage motion and queues instructions to the master I/O board (**D**). The master I/O board schedules and delivers each instruction to the appropriate channel I/O board (**E**) using the CAN bus, which in turn, sets focus (**F**), opens the remote-head camera’s electronic shutter (**G**), powers the LEDs (**H**), closes the shutter, and delivers the resulting image to the computing cluster (**J**), all with less than 1 ms of latency. Experiments require that the user assemble specimens dosed with beads into a conventional 96-well multiwell plate (**K**). The AHTM visits the entire plate in 7 steps (**L**). Graphics for (**B**) and (**C**) were obtained from openclipart (http://openclipart.org).

Figure 1 shows the overall structure of the AHTM main body (Fig. 1A), its support hardware (Fig. 1B–L), and its primary signal pathways. Twelve 40× objectives sit inside the traveling range of an XY translation stage (Ludl) which itself rests on three Z-stepper motors (Haydon-Kerk). The user configures an experiment by recording the desired system settings and details about plate contents as metadata in the user interface (UI;Fig. 1B) running on the master computer (MC;Fig. 1C). Interactive imaging for all twelve channels helps the user to optimize AHTM settings, which include imaging channels/mode, exposure times, FOV per well, and choosing to use autofocus.

In addition to serving as the UI, the MC provides system control for the XY translation stage, Z-stepper motors, and electronic backplane through Universal Serial Bus (USB) connections. When an acquisition begins for a FOV, the XY stage moves into the requested position above the objective array and simultaneously images within 12 wells of a Society for Laboratory Automation and Screening (SLAS) standard^35^ 96-well plate. Z-stepper motors minimize tilt and center the plate’s vertical position within the available focus range of each objective’s liquid lens.

The electronic backplane contains 14 boards in total. The single master I/O board (Fig. 1D) serves as the system ‘conductor’, orchestrating synchrony between itself and each imaging channel’s I/O board (Fig. 1E) by scheduling and sending commands though the controller-area network (CAN) bus. Both master and channel I/O boards have one microcontroller each (Piccolo TMS320F28035; Texas Instruments,). A motherboard (not shown) carries communication signals/messages between master I/O and channel I/O boards.

**Figure 2:**
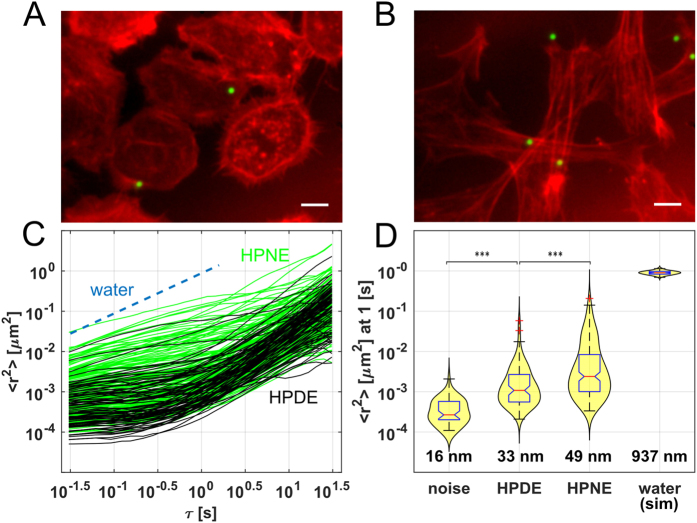
Imaging cells and PBR measurements in the AHTM. Imaging and example MSD data from HPDE and HPNE cultures. HPDE (**A**) and HPNE (**B**) cells imaged with the AHTM in the 490 nm and 575 nm channels and merged in ImageJ. 2 μm YG beads are shown in green and 568 alexafluor phalloidin stained F-actin in red. MSD vs τ curves measured by the AHTM are shown in panel **C**. HPNE (green) and HPDE (black) curves, selected for the anomalous diffusion model are shown, with a reference line for the calculated diffusion of similar particles in water. At τ = 1 s, the median of all curves are plotted, with summary statistics, in panel **D**. The expected median MSD and distribution for beads attached to the substrate (noise) and in water (simulation) are also presented as a reference along with RMS displacements in nanometers.

When an experiment begins, the MC sets the sample plate at the proper height in Z, compensates for tilt, and moves the XY stage to its first prescribed position above the objective array. Once the stage is in place, the master I/O board instructs each channel to set focus on its liquid lens (Fig. 1F). For the experiments described here, we configured the system to collect images by sending the following sequence of directives to the master I/O board: (1) open the camera’s electronic shutter (Fig. 1G), (2) power the LEDs (Fig. 1H) for the appropriate exposure time, (3) close the shutter, (4) save the resulting image to the computing cluster (Fig. 1J). The master I/O board relays this sequence to the channel I/O boards which then execute the instructions for the total number of frames.

Each 40× objective offers vibration-free focus across a 650 μm range (Supplementary Fig. 3A) by using a voltage-controlled liquid lens (Arctic 316, Parrot, Inc.). Applying voltage to the contact pin changes the electrowetting properties of the lens and the objective’s POF. Each lens required centering and insertion into its objective under cleanroom conditions and a contact pin installed through the barrel to conduct current to the lens (Supplementary Fig. 3B). Adding the liquid lens reduced the objectives’ NA from an initial 0.65 down to an estimated 0.45, a choice that sacrificed imaging resolution. Even so, benchmark testing proved the system still capable of returning accurate viscosities when imaging 200 nm beads in water at 50 ms exposures^22^.

Each channel contains two LEDs which serve as light sources that excite in the FITC, E-GFP (490 nm), and Texas red (575 nm) ranges. In addition to these standard fluorophores, the LEDS also strongly excite standard “yellow-green” (YG) and “red” fluorescent microbeads which are extremely bright, resist photobleaching, and are commercially available in a wide variety of sizes (Fluospheres, LifeTechnologies). Custom dichroic cubes (Edmund Scientific), designed and manufactured to fit into the tight spaces required, pass excitation light from the LEDs to the sample and emission light from the sample to its camera (Dragonfly, Point Grey). Each camera is instructed to collect an image when triggered by a signal generated on a channel’s I/O board. Images are transmitted via FireWire to a cluster of 12 computers. Once imaging is finished for a particular FOV, the AHTM translates the plate to a new position to image in a new set of 12 wells (Fig. 1K), where 8 translations visits all 96 wells (Fig. 1L).

Besides accommodating the tight packing the SLAS standard footprint required for the optics and cameras, the largest design challenge for the AHTM was strict and simultaneous control over focus, cameras, and illumination sources for all 12 imaging channels as well as XYZ stage translation. To address this challenge, we designed a custom-made embedded operating system called LEWOS (Launching Events With Optimal Synchrony) which provides microsecond level control for all 200 + real-time signals transmitted to and from AHTM hardware, with <1 ms latency. Using more than 12 objectives challenged the size constraints of the SLAS standard^35^ and using fewer inflated experiment duration.

Adding the liquid lenses presented two challenges. First, the magnification changed slightly with changes in POF. The POF is at its maximum distance at zero voltage, and experiences no observable change until V_RMS_ >30, where it becomes progressively closer to the objective as voltage increases. Supplementary Fig. 3C shows calibration curves that convert input RMS voltage and thus each POF into a Z displacement (black circles) and pixel distance (red line). A second complication is that the electrowetting lens exhibits hysteresis, where the same voltage approached from different directions shows slightly different POFs and magnifications. We therefore drive the lens from the low voltage regime to the desired focal POF every time a new focus is needed. For cell experiments, fibronectin-coated, 2 μm diameter YG microbeads were added to cell monolayers, given time to attach, and then non-adherent beads washed out. We were thus able to base our autofocus algorithm on finding the one POF with maximum intensity relative to the background (Supplementary Note 1). In practice this algorithm was performed on each cluster computer simultaneously. Once an objective establishes its POF, videos are taken at the maximum frame rate available for the bead type used. We acquired images for 2 μm YG beads at 33 fps for 60 s, a rate and duration that provided sufficient sampling of bead diffusion for constructing mean-squared-displacement (MSD) vs time window (τ) curves and calculating relative cell compliance^18^,^19^,^20^,^21^,^36^.

The AHTM typically generates ~1 TB of video during every hour of data collection. In contrast, bead trajectories, obtained by an automated version of Video Spot Tracker (CISMM.org), require trivial amounts of space^37^. However, to provide a means for auditing video data and still save space, we developed a compression algorithm that faithfully finds regions with motion and sufficient surrounding area to allow retracking, while storing the average image in background regions. We then use lossless H.264 compression to further reduce retained video file sizes by 99% without sacrificing tracking accuracy (Supplementary Note 2).

While we take data on cultures that are ~75% confluent, there are still spaces between cells where fibronectin-coated beads can adhere directly to the substrate. In addition, although we gently wash out unattached beads before data collection, some small fraction often remain diffusing through the sample space during each video. We therefore needed a method to eliminate the substrate-attached and freely diffusing beads from the analysis. We applied a Bayesian approach based on work that separated trajectories into models which correspond to elastic (N), Newtonian (D), confined (DR), or anomalous (DA) diffusive motion^38^,^39^. Previous work identifies anomalous diffusion as a motion profile for membrane mechanics and beads embedded within cells^36^,^40^ so we chose to restrict our analysis to trajectories the Bayesian approach labeled as belonging to the DA model with a posterior probability >0.5. Most trajectories in the population fit the DA model for all cell types (Supplementary Fig. 4). While we did see a marked increase in the prevalence in DR model selection between the DE and NE cell types, this model behavior never exceeded 10% of the total number of trajectories scanned. In our data, trajectories matching the anomalous diffusion model correlate with progression to the more invasive phenotype.

### Pancreatic Cell Cultures

Nearly all pancreatic cancer involves malignant transformation of pancreatic epithelial duct cells, and mutations in the *KRAS* oncogene are present in over 90% of metastatic pancreatic cancers^41^. We used HPNE cells as our model invasive cell line, comparing them to non-transformed HPDE cells. Figure 2(A,B) shows both cell types cultured onto a plastic-bottomed, 96-well plate and decorated with fibronectin-coated 2 μm YG microbeads. Actin filaments and cell boundaries were clearly delineated using a 500 ms exposure compared to 12 ms for bead images, showing the AHTM capable of co-locating beads with labelled cellular structures. Panel C plots MSD vs τ for a representative experiment where black (HPDE) and green (HPNE) lines mark each tracked bead and only those trajectories that fit the anomalous diffusion model are shown. HPNE bead trajectories are more heterogeneous, with a higher proportion of beads showing greater MSD values at most τ. Panel D provides violin plots for each experimental condition at τ = 1 s along with simulated conditions for water as a reference. Using van Elteren’s test (see Supplementary Note 3 and Supplementary Table 1), the data demonstrate a statistically significant increase in median MSD and RMS displacements between HPDE and HPNE cells (p_adj_ < 0.0001). Thus the AHTM combined with PBR techniques easily detects the difference in rheological properties between “normal” and transformed cells with a strongly invasive phenotype.

Studies recently published by the Wood laboratory describe a GOF library of pathway-activating lentiviral constructs and its application of discovering pathways that confer resistance to targeted anticancer therapies^42^. To assess whether the AHTM could detect more subtle changes in phenotype related to invasion, we chose 4 constructs from that library and tested for a change in invasion potential. The H-Ras (Ras pathway) and TGF-ß receptor-type I (TßRI; TGF-ß pathway) GOF constructs were chosen based on their known effects as an oncogene and in initiating TGF-ß signaling, respectively^31^,^33^,^43^,^44^,^45^. Myristoylated-Akt (PI3K/AKT/mTOR pathway) and BCL-2 (intrinsic apoptosis pathway) GOF constructs were chosen because of their known roles as genes promoting growth and suppressing apoptosis during cancer progression^25^,^26^,^28^,^29^.

**Figure 3:**
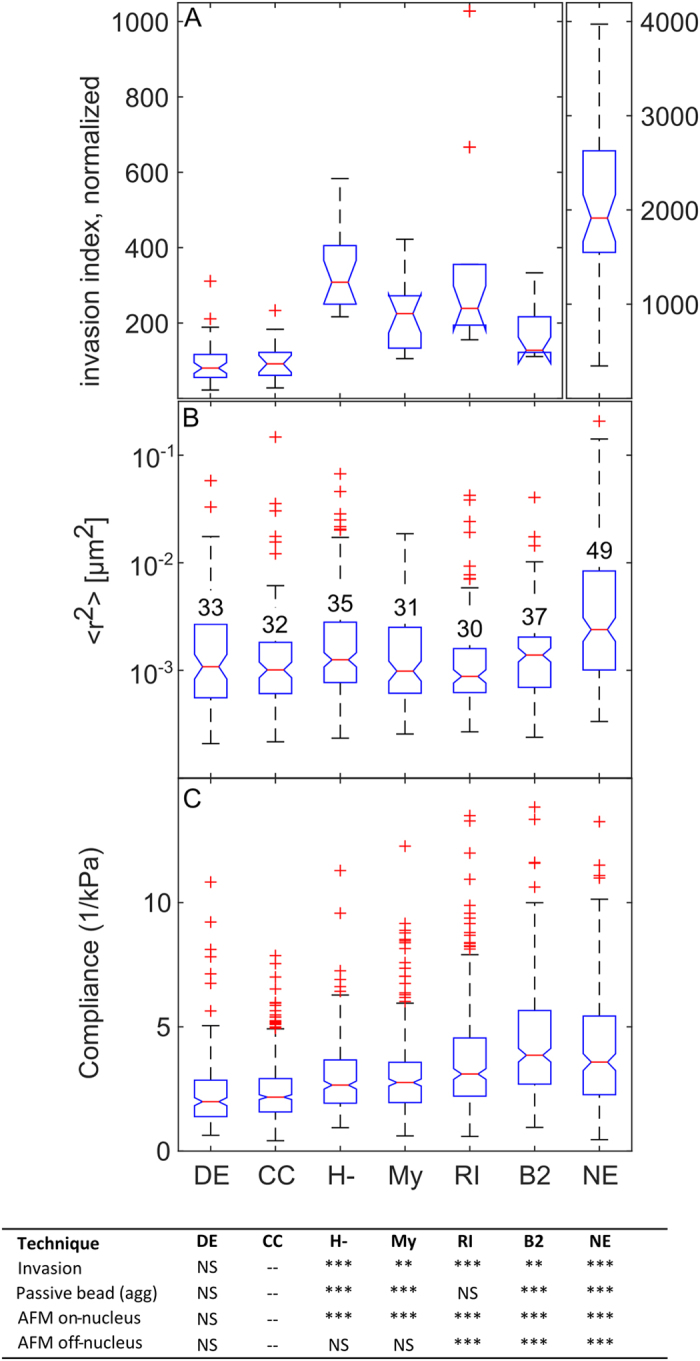
Effect of constructs on Invasion and Cell Compliance. (**A**) Notched boxplot showing results of invasion assay, where added constructs correlate with higher invasivity. (**B**) Median MSD and RMS displacements in nm at τ = 1 s for each construct, as assessed by the AHTM system for Plate 2. Results for the 4 separate 96-well plates used in this study are shown as Supplementary Fig. 5. (**C**) Median compliance data AFM measurements over cell nucleus. (**D**) Table of significance values for the four constructs and three comparison cultures shown in (**A**–**C**), along with off-nucleus AFM data (Supplementary Fig. 7), where *, **, and *** correspond to Bonferroni-corrected p-values p < 0.05, p < 0.01, and p < 0.001 respectively.

We first tested the effects of these constructs on invasion in stable expression HPDE cells, using the standard Matrigel invasion assay. In each case, the GOF was functionally validated in the stable cell lines using standard methods, the results of which are shown as Supplementary Figs 1 and 2. Figure 3A shows invasion indices for HPDE (DE), construct control (CC), H-Ras G12V (H-), Myristoylated-Akt (My), TßRI-T204D (RI), BCL-2 (B2), and HPNE (NE). Wilcoxon rank-sum tests were used to compare constructs and statistical significance was determined using the Bonferroni corrected p-values. While each construct increased invasion to an extent that was intermediate between CC and NE, all were significantly enhanced relative to CC (p_adj_ < 0.01).

Figure 3B shows data from the second plate run on the AHTM, where we compare MSD for the same conditions. We arranged the cells in the wells so that each objective viewed the same pattern of cell populations to minimize any bias introduced by differences in objective/camera pairs. Video data in the YG channel was collected for each cell treatment, and the bead motion analyzed for MSD vs τ. Each testing condition for the entire study contained data on an average of 525 beads across 192 FOV, i.e. approximately 3 beads/FOV or 1 bead for every 2 cells at 75% confluence. Results for individual plates are presented in Supplementary Fig. 5 while combined results are shown in Fig. 3B. The plots show the median MSD values and RMS bead displacements at τ = 1 s. We observe a ~16 nm (~30%) increase in the median RMS value between cultures at both phenotypic extremes (CC vs NE), and a ~5 nm increase between CC and H-Ras, myr-AKT, and BCL-2 and a lesser increase for TßRI. As seen in the individual plates (Supplementary Fig. 5), cellular response when expressing the TßRI construct was less consistent than other constructs. To further compare the results, we plotted the median MSD versus the median invasion index for each condition, normalized both against CC (Supplementary Fig. 6). Except for R1, which showed no significant change in MSD over CC, the data show that a higher MSD correlates with a higher invasion index. This inconsistency and the resulting lack of a statistically significant difference between TßRI and CC (summarized in Fig. 3D) may imply a more complicated effect of TßRI receptor expression.

To determine whether PBR results would correlate with more conventional, high precision/low throughput methodologies, we compared AHTM results to splits of the constructs using AFM. We used a 5 μm polystyrene bead attached to the AFM cantilever, and determined median compliances for each construct and controls for >25 cells of each type (Fig. 3C) (Supplementary Fig. 7). Wilcoxon rank-sum tests with Bonferroni-corrected p-values established statistical significances between constructs using AFM.

When inverted, the resulting median compliance values, which ranged between 2 and 4 1/kPa, correspond to a stiffness of ~500 Pa in the HPDE and CC cells, and to ~250 Pa in the HPNE cells. These stiffness values are consistent with other AFM studies of metastatic cells. Investigations of cultured breast ductal adenocarcinoma cells and pancreatic adenocarcinoma cells^10^,^12^ yielded stiffness ~500 Pa, while studies on breast cancer tissue showed stiffnesses in the 300–500 Pa range^46^. The constructs generally displayed intermediate compliances, except for BCL-2 expressing cells which returned a larger median compliance than even HPNE cells. The difference between BCL-2 and HPNE cells, however, was not statistically significant at the 0.05 level. In terms of statistical significance, we can summarize the data from each of the assays relative to the CC controls (Fig. 3D). PBR and AFM results agreed in direction and significance for H-Ras, myr-AKT, and BCL-2. For TßRI-transfected cells, conflicting results for MSD cells were reported on different plates (Supplementary Fig. 5 and Supplementary Table 2). Aggregate median values of the PBR assay were not significantly different from CC while the AFM showed a significantly increased median compliance both on- and off-nucleus (Supplementary Fig. 8B).

## Discussion

Our results show that the AHTM can readily distinguish non-transformed primary cells (HPDE) from cells that are far advanced in the transition to a more invasive phenotype (HPNE). Additionally, the system consistently identified more subtle changes in cellular mechanics generated from the expression of single-gene GOF constructs with the exception of TßRI. These constructs generally showed MSD values between those seen for HPDE and HPNE phenotypes. The same constructs caused only modest increases in invasion assay results and up to a two-fold increase in AFM compliance measurements.

We did not expect that all constructs used in this study would necessarily show a more compliant phenotype. Several constructs were chosen as possible indirect facilitators of metastatic progression. For example, My-AKT and BCL-2 expression promotes the ability of cancerous cells to multiply without undergoing apoptosis, but is not known to alter the mechanical phenotype of pancreatic cancer cells directly. The statistical significance found in both the PBR and AFM assays may indicate an important mechanical change that indirectly accompanies expression of these genes.

The TßRI construct produced the largest increase in invasiveness and the second highest increase in compliance as measured by the AFM, but gave inconsistent results in the PBR assay. Further work will be necessary to determine why the TßRI response using MSD data was not as consistent as seen in the other assays. Perhaps that particular construct was more heterogeneous in its expression than the others. If it were fully expressed in a small fraction of transfected cells, invasion assay and western blot results could still be strongly increased over controls. Such an explanation, however, would expect higher variability in RI results relative to the other conditions, which we do not see (Fig. 3C).

Figure 3D summarizes that, with the one exception, the assays performed consistently in the sense that increased compliance and invasion and levels of significance were in agreement for each treatment group. It is notable that agreement between AFM and AHTM measurements were largely consistent, even though they measure cell membrane compliance differently. The AFM applies nanonewton forces, indenting cells up to 1 μm and assessing compliance well into the cell body, including the cortical actin network^47^. In contrast, PBR probes the membrane and cortical actin cytoskeleton less invasively by assessing both the fluctuations of the cell as an active medium^48^, as well as its properties as a material responsive to sub-piconewton, thermal forces. Therefore, differences in the AHTM MSD across cell types potentially reflect both cell compliance differences as well as differences in the intensity of random uncorrelated motor-driven forces driving the bead. The agreement between AFM and AHTM measurements, in the direction and statistical significance of comparisons between the cell populations, is striking and suggests that AHTM results primarily reflect mechanical compliance. However, differences in ATP driven motor activity may play some part in observed differences in the AHTM data. Disentangling the contributions of compliance and active processes to the AHTM signal can be addressed through ATP depletion experiments^49^,^50^. This addition to the current protocol would enable the ability to independently correlate cell compliance and the overall intensity of motor activity within the cell with metastatic potential.

The AHTM is capable of performing many other HT screening assays in addition to measuring cell membrane compliance. For example, the Wood library was constructed to examine the effects of signaling pathways on the survival of cancer cells treated with anticancer agents. Programming the AHTM to discriminate between live and dead cells using standard fluorescent markers would create an even better assay that could rapidly screen for genes or pathways that affect drug sensitivity in addition to monitoring changes in apparent compliance. Such assays typically involve binning procedures to identify candidate hits^51^. Our results could likewise be summarized using bins related to statistical confidence (p_adj_ < 0.05) and reporting the extent of mechanical change across the 4 screens. Consistent mechanical changes would put H-Ras, myr-Akt, and BCL-2 into a high-confidence bin, where further testing using additional assays is well motivated. Similarly, inconsistent TßRI measurements place this condition into a lower-confidence bin, where further testing could be motivated given ancillary motivation.

It proved to be critical to develop a data analysis pipeline to filter out bead trajectories that are inconsistent with cell attachment, accomplished here by using a model-based Bayesian algorithm. By using two-color fluorescence and a new brightfield imaging mode, future studies with the AHTM could directly correlate a trajectory with contextual information about the cell underneath the bead. Such information as on-nucleus vs off-nucleus, distance from the cell edge, or other morphological analyses, will inform the variables that most affect each category of trajectory, as well as the state of the cell. These new types of data acquisition for the AHTM, coupled with our lossless compression and automated analysis, will facilitate further development of cell mechanics assays that probe the pathways responsible for many cancer phenotypes, as well as the effects of chemotherapeutic drugs.

## Methods

### Preparation of gain-of-function cultures of HPDE cells

DNA plasmids encoding for open reading frame (ORF) overexpression of the constructs of interest were prepared in lentiviral vectors. The constructs were obtained from Dr. Kris Wood (Martz *et al.*^42^). First, the 20 ng of the plasmid DNA for each construct was amplified in DH5alpha competent bacteria using heat shock and standard methods. After MiniPrep, the lentiviral DNA and packaging DNA-plasmids Δ8.9 and VSVG were transfected into 6 cm dishes of 293 T cells (ATCC), grown for 2 days, and the virus-containing supernatant harvested daily. The virus was concentrated using 100 K Amicon Ultra centrifuge filter (Millipore). The ratio for virus versus cell number is 100 μL virus/million cells.

HPDE cells from Dr. Ming-Sound Tsao (University Health Network; Toronto, Canada) were infected with the lentivirus containing the construct of interest in HPDE growth medium (keratinocyte serum free medium supplemented with EGF, bovine pituitary extract and antibiotic-antimycotic), incubated for 48 hr, and then selected for infected cells with 1 μg/mL puromycin in HPDE growth medium. Whole cell lysates were subjected to 10% SDS-PAGE and total proteins were transferred to PVDF membrane where they were tested with the appropriate antibodies.

### Validation of activating pathway component expression in HPDE stable cells

Stable transgene expressions were confirmed by western blot (myr-AKT, H-Ras, BCL-2) and immunofluorescence (TßRI) as shown in Supplementary Figs 1 and 2. The numbers below each column are the percent of control levels, while the antibodies used to probe the blots are listed to the left of each column. Supplementary Fig. 1a,b show an increased expression of myr-AKT and H-Ras in transfected cell cultures, while Supplementary Fig. 1c shows that the BCL-2 expressing cells are able to block the apoptosis induced by gemcitabine, as evidenced by greatly reduced levels of caspase 3. TßRI expression was verified by highly increased levels of p-SMAD2 presence in transfected cultures (Supplementary Fig. 2).

### Focus Control with the Liquid Lens

The objectives lenses (UKA MO-0040 40×, 0.65 NA; (Universe Kogaku America) were retrofitted with voltage-controlled liquid-meniscus lenses, Arctic 416SLV3 (Varioptic, FR), to provide rapid individual focus control without mechanical vibration (Supplementary Fig. 3B). The liquid lenses fit inside the UKA inner barrels, and provide −12 to +18 diopters of optical correction to the nominal 4.33 mm effective focal length of the objective over an AC-controlled voltage range of 30 to 60 V_rms_. That voltage is generated using a Coilcraft 20X transformer, LPR6235, with its secondary resonated by a 1 nF capacitor + parasitics. This transforms a 5 V pulse-width-modulated signal to a 50 kHz sine wave over the required voltage range. Cross-talk between channels is an issue, requiring careful shielding of the 50 kHz signals as well as operating all twelve channels in phase lock.

Accurate and stable focus control requires a closed-loop feedback system for each channel. This is implemented using a Texas Instruments digital signal controller, TMS320F28335, with on-board analog to digital converters and pulse width modulators. The liquid lenses AC voltage is scaled, then synchronously sampled at well above Nyquist rate, and its RMS voltage compared to a digital control signal to calculate a feedback to the pulse width modulator. The pulse width modulated signal is amplified by an external power transistor which drives the resonated transformer, thereby closing the control loop.

### Calibration of Magnification with Focal Plane

As shown in Supplementary Fig. 3, applying a voltage to the liquid lens changes the POF towards the objective face (Supplementary Fig. 3A) but also causes an incremental change in magnification. The change in scale with voltage was determined for each objective, using fluorescent beads embedded in several mm thick silicon rubber (polydimethylsiloxane (PDMS)) within a 96 well plate. Autofocus (Supplementary Note 1) finds a plane with beads in focus, and the Master computer moves the XY stage known 3 μm distances in a sequence of 100 steps in random directions while recording video. This sequence of steps is used to calculate a first-order approximation of the scaling factor, which is then further refined by displacing the target bead across the entire field-of-view corner-to-corner. To acquire the same factors as a function of plane of focus, the system repeats this procedure across the full range of voltages (0 – 65.5 V) applied to the liquid lens. A scaling factor and an error are reported at each voltage. For the analysis used in the present paper, the scaling factors were close enough (<5% max error) to each other, that the mean curve was used for all channels.

### Plating of cells and preparation of microbeads for multiwell experiments

Cells were plated in their preferred medium (HPNE cells in RPMI medium in 5% FBS, antibiotic-antimycotic) at a density such that they achieved 60–80% confluence by the next day (5000–7000 cells/well depending on cell type). They were plated such that all cell type/treatments were assessed by each objective, to eliminate any bias caused by slight differences in the scale and resolution between objectives. Two micron diameter yellow-green fluorescent carboxylate microbeads (Life Technologies), were prepared by incubating 10 μL of stock beads in 1 mL 10 μg/mL human fibronectin (gift from Keith Burridge, UNC) in sterile phosphate buffered saline (PBS) for 60 min at room temperature with rotation, centrifuged to remove unbound fibronectin, washed in PBS and re-centrifuged. Beads were resuspended in 0.5 mL PBS, and aggregates broken up by 3 passes through a 26 gauge needle syringe combination. Equal volumes of diluted beads were added to each well with a multichannel pipetter, incubated for 90 min at 37 °C to allow adhesion to the cell surfaces, and then unattached beads were removed and fresh, pre-warmed media added. The plates were sealed with silicone tape, and data acquisition begun within 30 min.

### Invasion assay

Matrigel invasion assays, and transwell migration assays were performed using standard protocols. Cells were trypsinized and seeded at a density of 50,000 cells per well on either Matrigel-coated (BD Biosciences) or uncoated (Corning) Transwell filters in a 24-well plate and allowed to invade for 12 hours toward 10% FBS in the lower chamber. Cells invading and migrating through the Matrigel-coated and uncoated filters, respectively, were stained with Three Step stain (Richard-Allan Scientific). Each filter was counted in its entirety with four 10× fields, and invasion or migration was quantified as fold increase relative to control.

### Atomic Force Microscope Measurements

The stiffness of all human pancreatic cells and variants were measured using an MFP-3D Bio AFM (Asylum Research, Santa Barbra, CA). This is the standard AFM combined with an inverted optical microscope (IX71, Olympus) to enable precise positioning of the AFM probe over the desired region of the designated cells. The day before AFM experiments, cells were plated in their preferred medium onto fibronectin-coated glass coverslips. On the day of AFM experiments, the samples were rinsed with fresh medium to remove loosely attached cells. For all AFM measurements, cell imaging was completed with bright-field optical microscopy (cells were not fluorescently labeled).

A 5 μm diameter YG fluorescent polystyrene bead (Corpuscular, Inc; Cold Spring NY) was attached with a UV-curable adhesive (Norland Optical Adhesive no 81, Norland Products New Brunswick, NJ) to each TR400PSA (Olympus) silicon nitride cantilever. The fluorescent bead provides two functions: (1) a large uniform bead-cell contact area during indentation measurements avoids puncturing the cell membrane and (2) allows precise positioning of the bead relative to the area of interest on the cell. Prior to and following force measurements on a cell, the cantilever spring constant (nominal 0.02 N/m) was determined in buffer using the built-in thermal calibration software in IGOR/Asylum Software to ensure stability of force measurements throughout the experiment.

With the aid of the inverted optical microscope and 2D micromanipulation stage below the AFM head, the beaded AFM cantilever was placed above either the center of the cell nucleus (“on-nucleus”) or approximately 5 μm to the side of the nucleus (“off-nucleus”). The cantilever was moved at a velocity of 5 μm/s downward toward the cell until a trigger force of 1 nN (on-nucleus) or 0.5 nN (off-nucleus) was reached. The cantilever was then retracted at the same rate. For most cells, this was equivalent to ~1 μm of indentation on-nucleus and ~0.5 μm off-nucleus. Smaller indentations were used off-nucleus due to the smaller thickness of the cell at those locations. For each cell, ten force curves were collected at each location, separated by a 30 s dwell away from the cell surface. Force measurements were acquired for a minimum of 30 cells for each cell type over the course of two days of experiments.

Calibrated cantilever deflection and piezo displacement data collected were converted to produce force vs. indentation curves. Force was determined according to Hooke’s Law, *F* = *k*Δ*d*, where k and d are the spring constant and deflection of the cantilever, respectively. Cell indentation was determined from the difference in piezo motion displacement and cantilever deflection. The force-indentation curves were analyzed using a custom MATLAB procedure to calculate the cell stiffness using Hertzian contact mechanics^52^,^53^,^54^. Briefly, the data was assumed to take linear form for the pre-contact data, and the Hertzian form for the post contact data. The contact point is determined by finding the fit with the minimum RMS error. The Young’s modulus was then extracted from the least squares fit of the Hertz model to the post contact region. For a spherical tip, the following relationship for Hertz model was used:


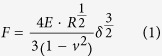


where R is the radius of the spherical tip, δ is indentation, and ν is the Poisson ratio (set to 0.5), and E is the Young’s modulus of the cell.

## Additional Information

**How to cite this article**: Cribb, J. A. *et al.* An Automated High-throughput Array Microscope for Cancer Cell Mechanics. *Sci. Rep.* **6**, 27371; doi: 10.1038/srep27371 (2016).

## Supplementary Material

Supplementary Information
