# Supplementary material for: An Automated High-throughput Array Microscope for Cancer Cell Mechanics
Source: Sci Rep. 2016 Jun 6;6:27371. doi: 10.1038/srep27371 (PMC4893602; doi:10.1038/srep27371)
Supplement: Supplementary Information [file srep27371-s1.pdf]

Supplementary Information

An Automated High-throughput Array Microscope for Cancer Cell Mechanics

Jeremy Cribb[1]*, Lukas D. Osborne[1]*, Kellie Beicker[1], Matthew Psioda[3], Jian Chen[4], E. Timothy O'Brien[1], Russell M. Taylor II[2,1], Leandra Vicci[2], Joe Ping-Lin Hsiao[2], Chong Shao[2], Michael Falvo[1], Joseph G. Ibrahim[5], Kris C. Wood[6], Gerard C. Blobe[4], Richard Superfine[1]

[1]Department of Physics and Astronomy, UNC-Chapel Hill, Chapel Hill, NC, [2]Department of Computer Science, UNC-Chapel Hill, Chapel Hill, NC, [3]Department of Biostatistics, UNC-Chapel Hill, Chapel Hill, NC, [4]Department of Medicine and Pharmacology and Cancer Biology, Duke University Medical Center, Durham, North Carolina, [5]Department of Biostatistics, The Lineberger Comprehensive Cancer Center, The University of North Carolina School of Medicine, Chapel Hill, North Carolina, United States of America, [6]Department of Pharmacology and Cancer Biology, Duke University, 450 Research Drive, Durham, NC 27710, USA, *contributed equally to the work

Supplementary Note 1: Autofocus Algorithm

Autofocus in our system is based on finding the focal plane that exhibits the maximum relative intensity of fluorescence in the channel with adhered volume labeled microbeads. The system starts at zero voltage to the liquid lens (longest focal length) then steps through the focal range in “coarse” mode, using 2.6 um steps. At each focal plane it computes a smoothed image S as

$$S_{ij} = P_{ij} - 0.25(P_{up} + P_{down} + P_{left} + P_{right}) \quad (1)$$

where i and j denotes indices for image rows and columns respectively. For each pixel intensity P_{ij}, P_{up}, P_{down}, P_{left}, and P_{right} are the intensities ten pixels away from each P_{ij} in the four cardinal directions. S_{ij} is computed for each pixel in each focal plane, except for those covered by a 10-pixel wide image border. For each frame, the intensity values of the 3% of pixels with the highest values are averaged, and the median for the entire image is subtracted from that average. The intensity score, F, for each frame is thus calculated as:

$$F = Mean(highest\ 3\%) - Median(entire\ image) \quad (2)$$

The best focal plane corresponds to the plane with the highest F value. After scanning through the entire z space with coarse steps, the liquid lens is set back to a position 19.5 um “higher” (lower voltage) than the best focused plane found in coarse mode. Scanning then repeats using steps of 0.65 um each time, to determine the best focused plane. The finer scanning stops when the intensity score dropping significantly in two consecutive images. The algorithm recognizes the optimum plane, and then again goes to 0 voltage, and proceeds to that plane directly. If no plane shows a significant intensity score, scanning continues until it completes the scan and reports that there is no object. That channel then waits for the others to complete and the system has moved to a new location.

Supplementary Note 2: Lossless Compression

When acquiring video from 12 cameras at 50 FPS, ~1 TB of data is generated during each hour of operation. We therefore needed a method for compressing the data, while preserving enough of the information to re-track our beads to check errors. Because we only care about the position of a small number (usually less than 10 per FOV) of bright beads against a dark background, and these beads are attached to the cell membrane (so they are not translating large distances between frames) we approached compression with a goal of preserving the information in the pixels near each bead, to allow fully effective re-tracking, while and at the same assigning each background pixel the time-averaged intensity value for that pixel for that video. This plus standard H.264 compression provided highly efficient compression of the background while losslessly preserving the information around the beads. This procedure results in ~100x reduction in storage size without reducing the accuracy of retracking. In practice, tracking of bead position/frame is carried out with the uncompressed videos, and immediately after completion of an experiment, and then the bead positions are re-tracked using the compressed videos. If the results match, the original videos are deleted and the compressed videos stored.

The details of the compression method are described elsewhere [1]. Briefly, the algorithm depends on distinguishing between foreground and background within each frame. For microscopy video, noise is understood to be independent between neighboring pixels, whereas blurring (convolution with the point-spread function (PSF)) will spread the brightness and intensity changes of an actual object from one pixel to its neighbors [2]. We therefore used a measure of the correlation of brightness changes between each pixel and its eight neighbors (Pearson's correlation coefficient [3]) and applied a threshold to each frame to keep those pixels whose intensity changes were correlated with their neighbors. Because even independent random variables have nonzero correlations, a number of pixels will be falsely labeled as foreground. These pixels are likely to be spread evenly across the image, whereas true foreground pixels will be grouped into clusters that are at least as large as the main lobe of the PSF. To remove these false positives, the binary segmentations are refined by the mathematical

morphology erosion operation. Because accurate tracking requires pixels in the area near each bead, the resulting set of foreground pixels is dilated to include pixels that are close enough to affect analysis (this radius is dependent on the parameters of the analysis algorithm). Using the refined binary segmentation, the original video has each pixel in its background regions replaced by that pixel's time-averaged value. This removes noise, which makes the video more suitable to be compressed by a common lossless compression technique. Here we choose to use lossless H.264 compression. To verify that the compression had no impact on analysis, the compressed video is processed by the same analysis pipeline to make sure the results match those of the original video.

Supplementary Note 3: Statistical methods for Data Analysis

All trajectories that had posterior probability ≥ 0.50 of matching the DA model were included in the statistical analysis of MSD. We performed a single analysis combining all four plates for the primary analysis. Due to the possibility of batch effects affecting the MSD values for each plate, we performed an analysis that stratified by plate using van Elteren's test [4], which is an extension of the Wilcoxon rank sum test (a.k.a. Mann-Whitney test) that allows for a stratification variable. The results are included in Supplementary Table 1. Both the raw p-values and adjusted p-values (adjustment for multiple comparisons using a step-down procedure due to Holm [5]) are included for the purposes of comparison. All significant results remain significant even if the more conservative Bonferroni correction is used.

We further performed several sensitivity analyses to assess the robustness of our statistical conclusions to the procedure used to analyze the data in the primary analysis. Our sensitivity analyses were designed to address potential biases that were not accounted for in the primary analysis. First, we acknowledge the possibility that objectives could perform differently despite our calibration efforts. While our experimental design ensured that each objective should follow some beads for each construct, there is no guarantee that the number of beads for each construct followed by an objective will be the same. In fact, we observed instances where no beads for a particular construct passed our quality control measures leading to a marked imbalance in sample size within an objective across

constructs. In these cases, a bias due to differential objective performance could occur. To address this, we performed two sensitivity analyses. First, we again performed van Elteren's test but stratified by plate and objective instead of just plate. This approach essentially discards data from the objective for that plate run unless there is data from both constructs being compared. Hence, although this approach is conservative in terms of bias control, the method makes very inefficient use of our data given the imbalances we have observed. Secondly, we used a Box-Cox transformation [6] with $\lambda = -0.325$ to transform the scaled MSD values so that the assumptions of a normal linear model were more reasonable than for the log-transformed MSD values (which showed a marked departure form normality). Using the Box-Cox transformed MSD values, we fit a linear model with main effects for plate, objective, and condition (allowing separate error variances for each objective) and performed comparisons of each construct to CC using normal theory (Supplementary Table 1). In both cases we find good agreement with the primary analysis results and it is conceivable that the lack of agreement between the two van Elteren's tests is due to a lack of power of the sensitivity analysis test procedure.

Even after applying the Bayesian classification algorithm as a filtering step, the final analysis dataset is contaminated to some degree. For examples, small numbers of beads clusters are tracked in lieu of single beads and it is plausible that MSD values would differ between such clusters and individual beads. Thus, if imbalances in the number of contaminants is not similar across constructs, biases could occur. We collect several quality control variables, including area of the bead (based on the initial image) as well as a sensitivity score. The quality control variables are predictive of contaminant cases such as bead clusters (bead clusters have larger areas than single beads). Both quality variables appear to have an obvious and non-linear relationship with MSD in our dataset (data not shown). To investigate the impact of these quality control variables on our statistical conclusions, we created factor variables for area and sensitivity where the levels of the factors corresponded to the deciles of the observed distributions of the quality control variables and adjusted for these variables in the same linear model described above. While both factor variables were highly predictive of transformed MSD (unadjusted p-values both <0.0001), the ultimate conclusions about construct

differences did not change. (Supplementary Table 1). In general, all of the sensitivity analyses that we performed show strong agreement.

We were also interested in assessing the reproducibility of the PBR results from individual plates. Supplementary Table 2 presents the p-values from a Wilcoxon rank sum test comparing each other construct to CC with appropriate p-value adjustment performed separately for each plate. A sensitivity analysis that uses van Elteren’s Test (stratified by objective) is included alongside.

In three of the four plates we are able to detect significant differences in MSD between NE and CC control after adjusting for multiple comparisons (within the plate). Interestingly, the one plate where this is not so is plate three where DE appears to differ significantly from CC, unexpectedly. This suggests a genuine type I error may have occurred for plate three (the CC sample indicates a median MSD that is not indicative of CC in general), that an issue with data collection may have occurred, or there is some uncontrolled bias that confounds the statistical results. Note that the aforementioned result does not hold for the more conservative analysis that stratifies by objective (after adjustment for multiple comparisons) though we are still able to detect CC versus NE differences using that approach. More experiments are planned to fully investigate the reasons for variability in the results based on individual plates.

Supplementary Figure 1: Western Blots

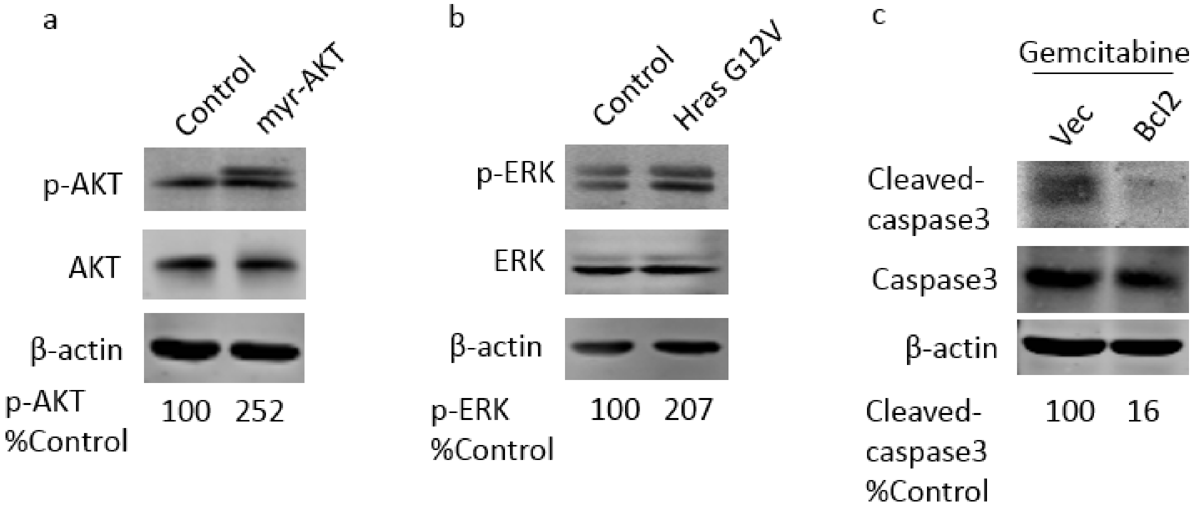

Supplementary Figure 1: Functional validation of Bcl2, and Gain-of Function Mutants of Akt (myr-AKT) and H-Ras (HRasG12V). HPDE cells were infected with lentiviruses expressing control vector or a) myr-AKT, b) HRasG12V, or c) Bcl2 for 48+h and selected with puromycin as described, followed by extraction, 10% SDS-PAGE, total protein transfer to PVDF membrane, and probing with the indicated antibodies (left of each blot). a) Activation of Akt signaling by myr-AKT is indicated by increased phosphorylation of Akt (p-Akt), b) Activation of H-Ras signaling by HRasG12V is indicated by increased phosphorylation of its downstream target, ERK(p-ERK), c) Bcl2 transfected cells were treated with gemcitabine (50 µg/ml) for 16 h before extraction. Functional increase in Bcl2 expression is indicated by a decrease in gemcitabine-induced apoptosis, demonstrated here by decreased cleavage of caspase3. Quantitation of these data, normalized to %-control is indicated below.

Supplementary Figure 2: Cell Transfection & Staining

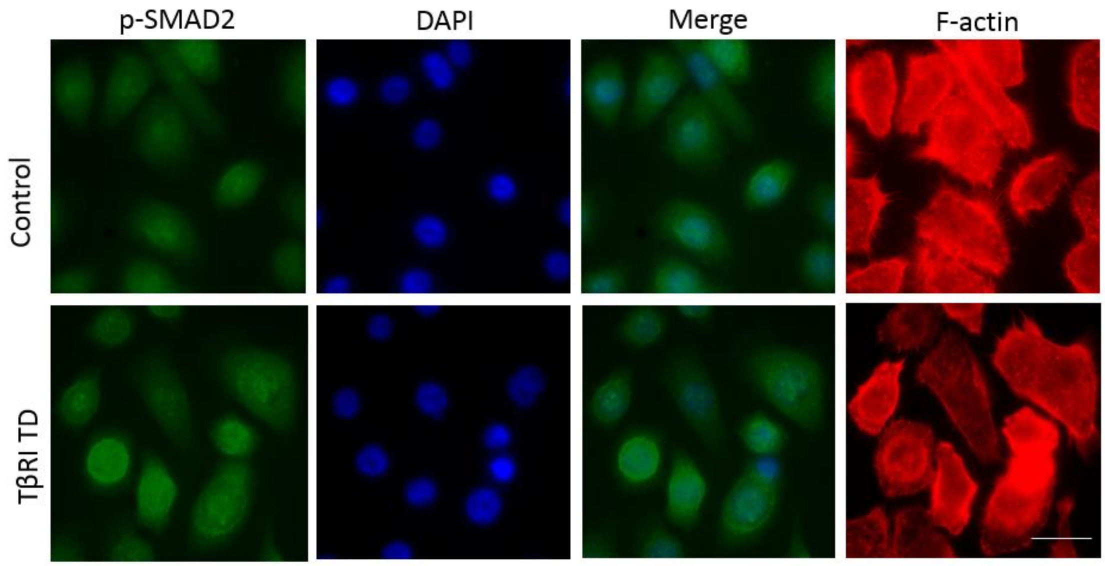

Supplementary Figure 2: Functional validation of the Gain-of Function Mutant of TβRI (TβRI- T204D). HPDE cells were infected with lentiviruses expressing control vector or TβRI- T204D for 48+ hr and selected with puromycin as described. The cells were then seeded on coverslip glass overnight, fixed with 4% paraformaldehyde and immunostained with the indicated antibody. Nucleus was counterstained with DAPI (1 µg/ml) and filament actin was stained with phalloidin (0.1 µg/mL). Images were taken under a Nikon fluorescence microscope and quantified using a Li-cor Odyssey v3.0. Activation of TGF-β signaling by TβRI- T204D is indicated by an increase in phosphorylation and nuclear localization of Smad2 (p-Smad2). Scale bar is 50 µm.

Supplementary Figure 3: Liquid Lens Schematic and Calibration

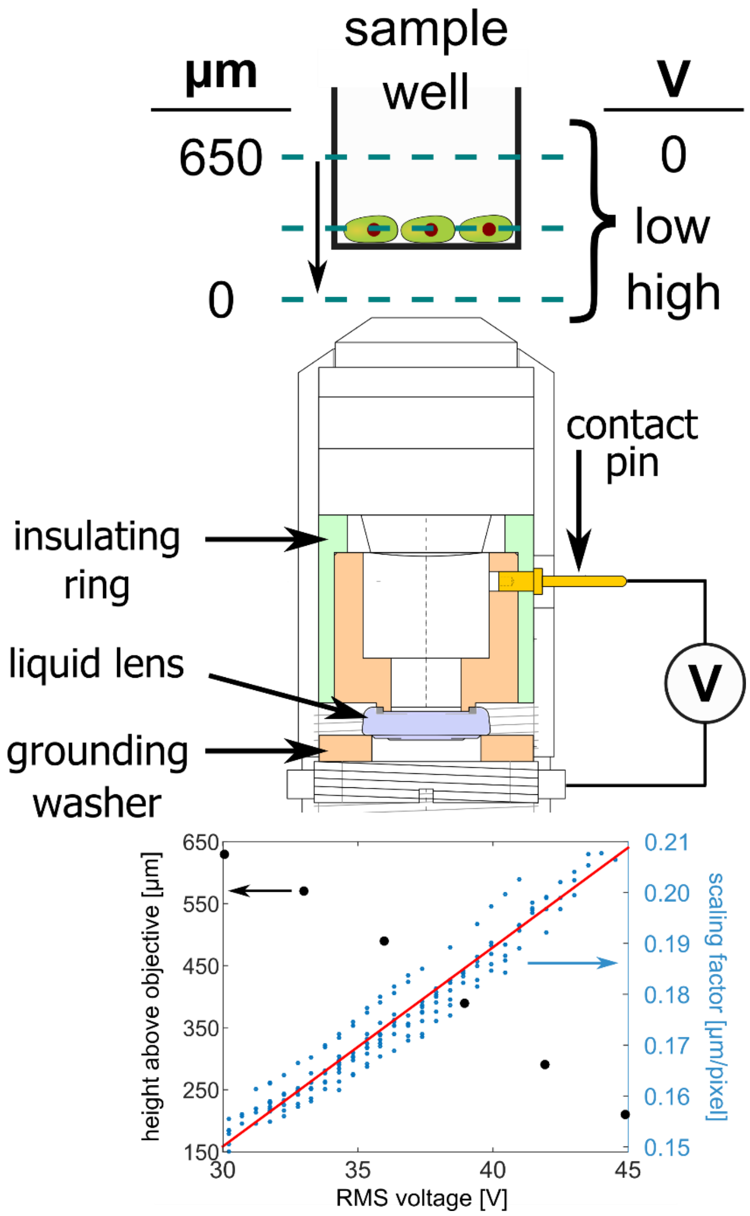

Supplementary Figure 3: The liquid lens, Arctic 416SLV3 (Parrot, FR), uses electrowetting to change the plane of focus for channels in the AHT system, which is at its highest point within the 96-well plate at zero volts and travels towards the objective lens as the voltage increases (A). Changing focus does not occur until the applied RMS voltage exceeds 30 V. Changing focus changes the magnification, shown as a calibration curve in (C).

Supplementary Figure 4: Bayesian Model Selection for Passive Bead Measurements

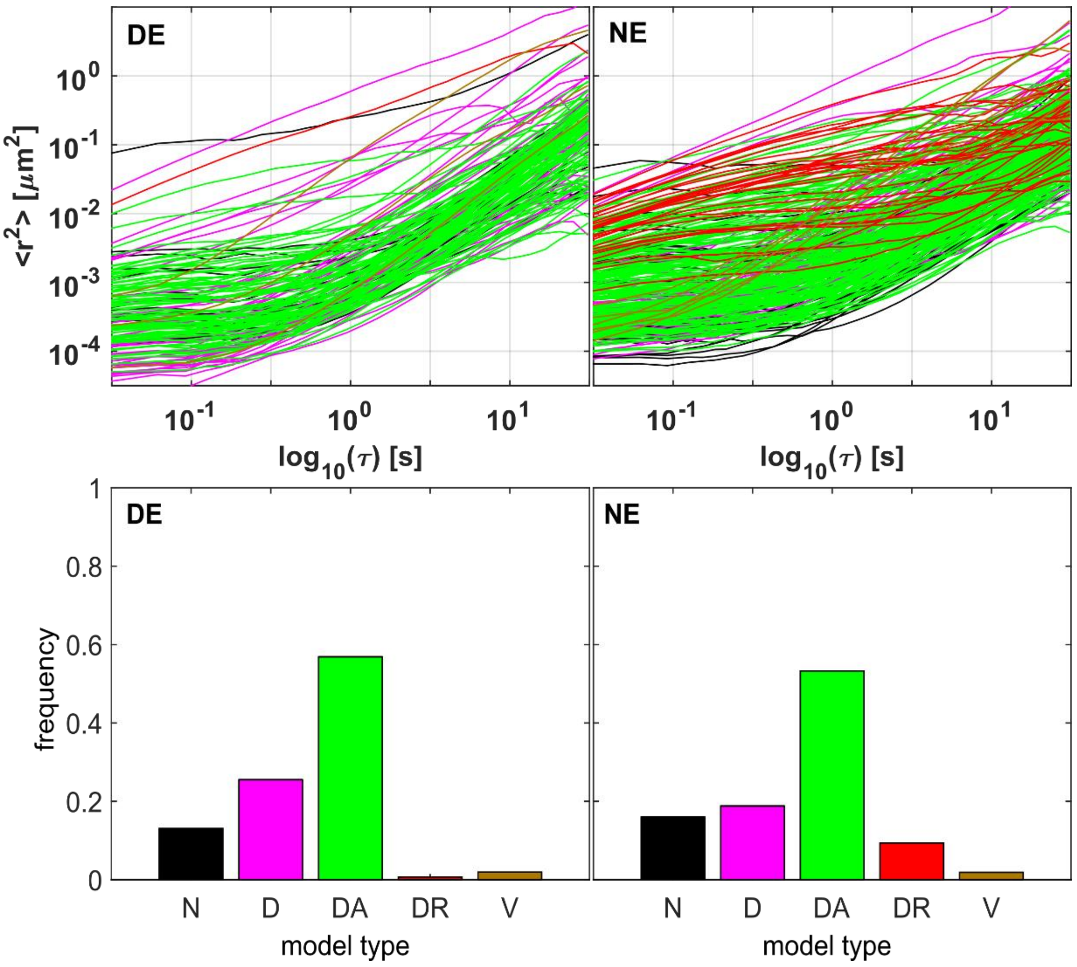

Supplementary Figure 4: (Top) Full MSD trajectories for 2 µm fibronectin-coated fluorescent beads attached to cultures of HPDE and HPNE cells, at all time windows assessed by the AHT system. The trajectories are each color-coded to correspond to the best-fit model. (Bottom) Frequency of model selection for data displayed at top. Data shown in black correspond to the elastic model (N) which describe beads presumably attached to the plastic substrate. Purple lines (top) and bars (bottom) designate unattached beads that simply diffuse (D) in the surrounding buffer. The confined diffusion model (DR), shown in red, correlates with trajectories that reach a plateau at longer τ. The most prevalent trajectories (green) fit the anomalous diffusion (DA) model, where the slope of <r2> < 1 and whose curves do not roll over at longer time windows. We report our data in Figure 2 as the median

values of all the DA model trajectories at the τ=1 s. The more invasive HPNE cells exhibit a wider range of behavior with respect to the diffusion model than the HPDE cells. Median MSD values for HPNE cells are also higher for bead trajectories that match the DA model. Including DR model behavior would increase this value and further separate the distributions between both cell types.

Supplementary Figure 5: Passive bead data on Individual Plates

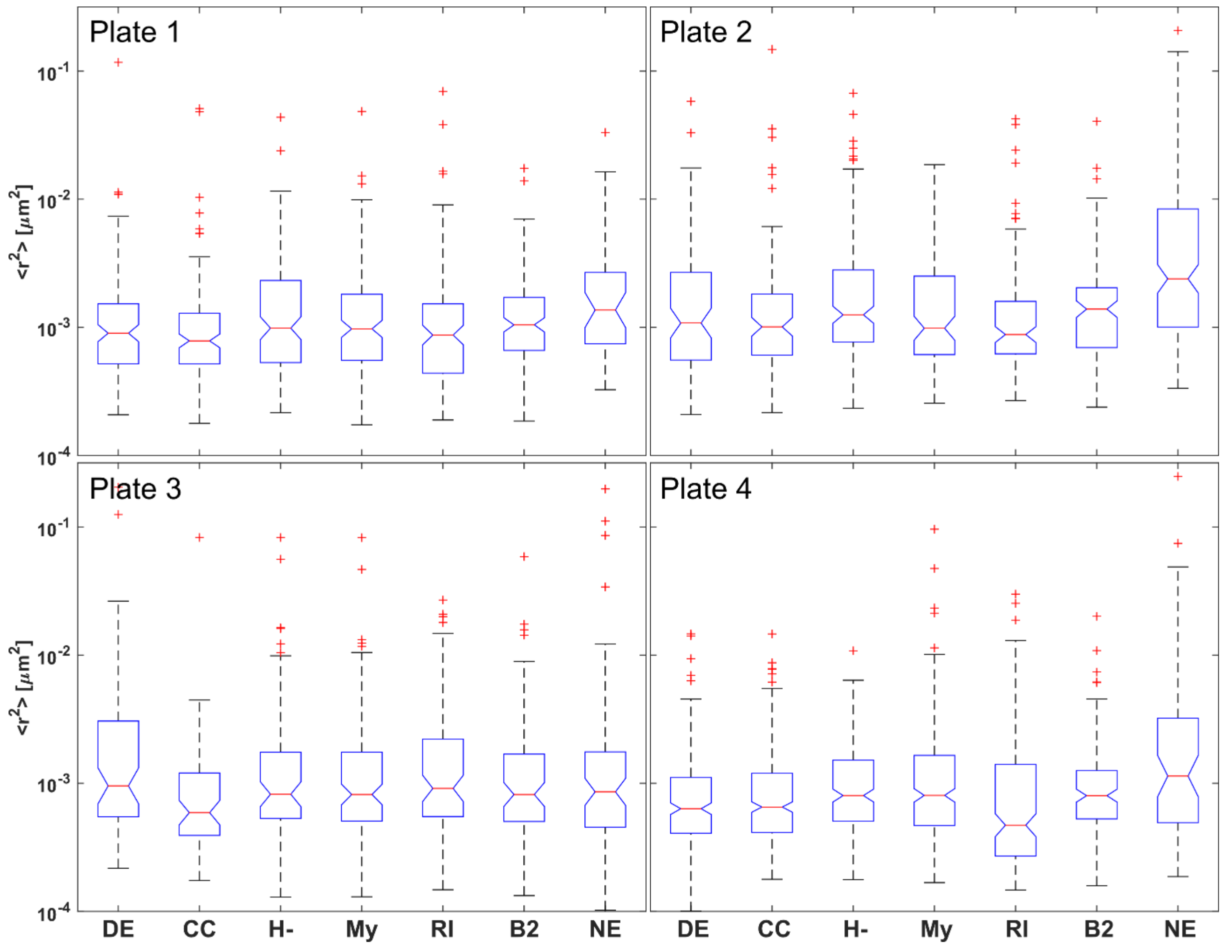

Supplementary Figure 5: Summary data for the 4 individual 96 well plates that are reported as an aggregate in Figure 3B. We tested significance relative to the CC (construct control). DE are the parent, non-transformed HPDE cells without any transfection. H- designates cells transfected with the gene for H-Ras, My = myristoylated-AKT, R1 = TßRI, and B2 = Bcl2. HPNE are the h-TERT immortalized, nestin and K-Ras expressing pancreatic ductal epithelial cells that are highly invasive.

Supplementary Figure 6: MSD vs. Invasion Assay

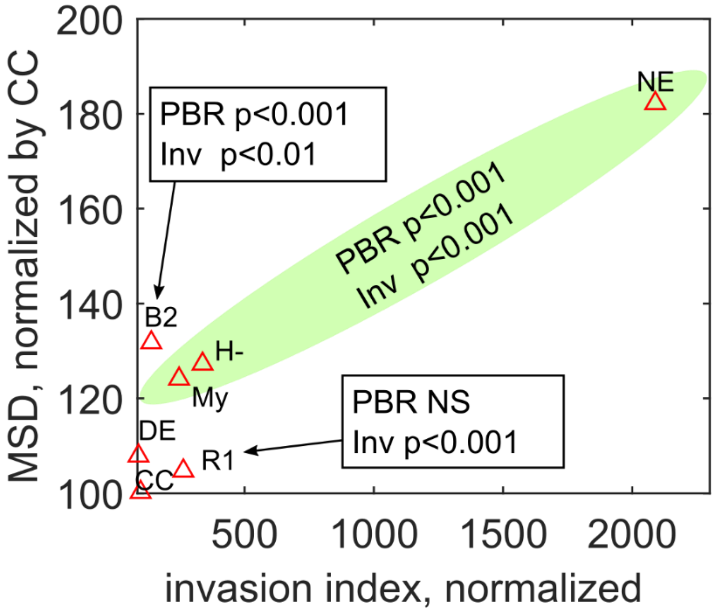

Supplementary Figure 6: Plot of the median MSD versus the median invasion index, normalized relative to CC (control construct), for each condition. Plot shows a correlation between higher MSD and higher invasion index with most significant region for passive bead rheology and invasion measurements indicated in green. Boxed labels, for B2 and R1 show the significance for both techniques.

Supplementary Figure 7: AFM Experiment Design

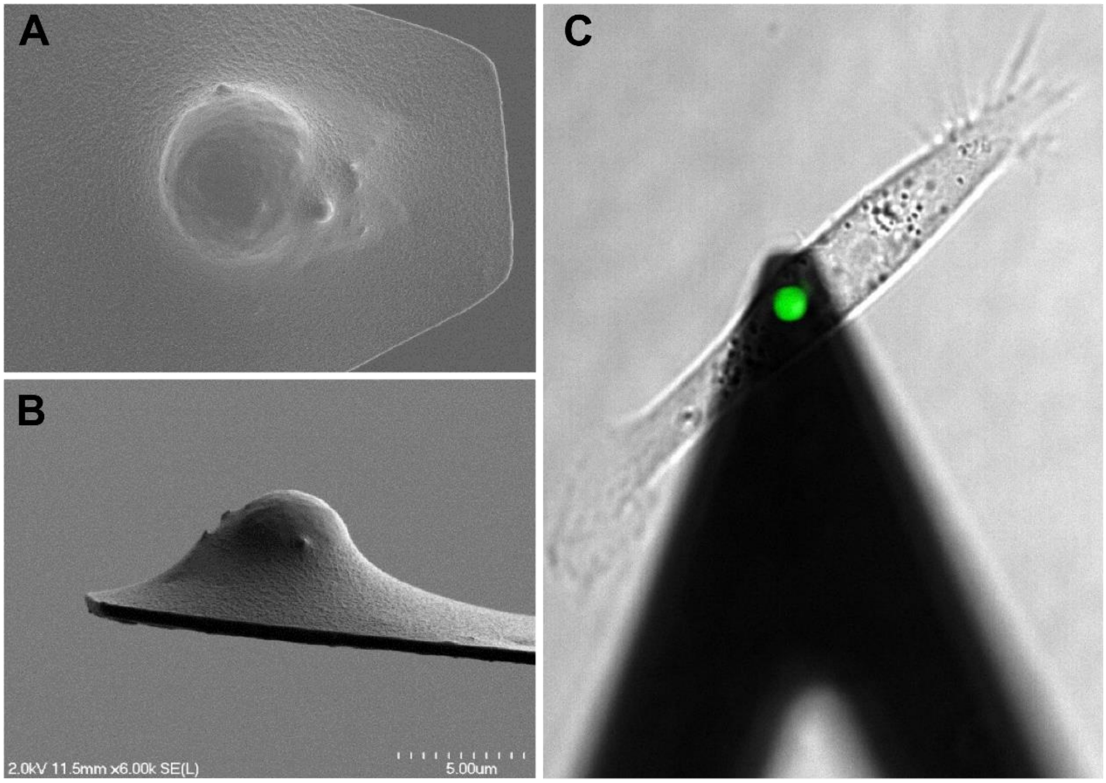

Supplementary Figure 7: Scanning electron microscope images top- (A) and side-view (B) of 5 μm bead attached to TR400PSA pyramid tip. (C) Bright-field and fluorescence images overlaid to show the alignment of YG fluorescent bead over HPDE cell.

Supplementary Figure 8: AFM Force Curve Analysis

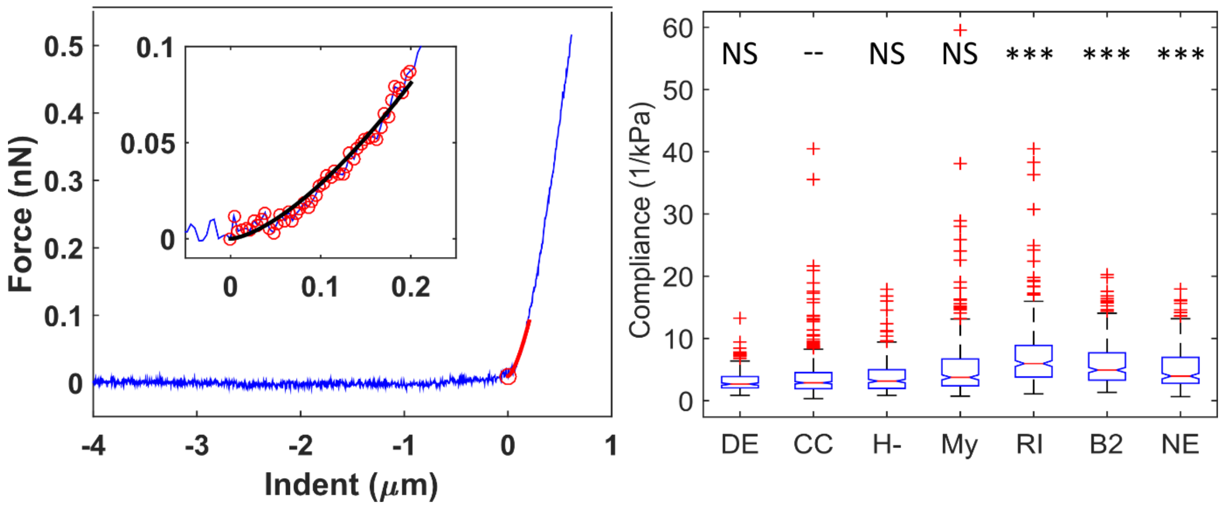

Supplemental Figure 8: (Left) Force-indentation curve acquired over the nucleus of a CC cell. The larger plot shows the entire force-indentation curve (blue), contact point determined by custom MATLAB code (red circle) and the Hertz model fit (red line) for 200 nm indent. The inset shows a detailed view of the Hertz model fit (black) with an elastic modulus of 322 Pa to the data points shown in red. The units on both the inset and main axes are the same. (Right) Compliance measurements for force curves acquired one bead diameter away from the nucleus on each cell type.

Supplementary Table 1: Statistical Significances for Constructs in AHTM

	van Elteren's Test with Plate Stratification		van Elteren's Test with Plate + Objective Stratification		Box-Cox Transformed Linear Model		Box-Cox Transformed Linear Model + Area Adjustment + Sensitivity Adjustment	
**Construct**	p	p_{adj}	p	p_{adj}	p	p_{adj}	p	p_{adj}
B2	<.0001	<.0001	0.0024	0.0118	0.0003	0.0013	0.0018	0.0070
DE	0.2560	0.5119	0.6534	1.000	0.2077	0.4154	0.3717	0.7433
H-	<.0001	<.0001	0.022	0.0878	0.0004	0.0018	0.0004	0.0019
My	0.0004	0.0011	0.0784	0.2351	0.0091	0.0272	0.0159	0.0476
NE	<.0001	<.0001	<.0001	<.0001	<.0001	<.0001	<.0001	<.0001
R1	0.5387	0.5387	0.8773	1.000	0.9842	0.9842	0.8807	0.8807

Supplementary Table 2: Statistical Significances for Individual Plate Runs

		Wilcoxon Test		van Elteren's Test with Objective Stratification	
Plate	Construct	p	p_{adj}	p	p_{adj}
1	B2	0.0121	0.0606	0.0408	0.2041
1	DE	0.2918	0.5836	0.5413	1.0000
1	H-	0.0429	0.1715	0.1087	0.4349
1	My	0.0761	0.2282	0.3004	0.9012
1	NE	0.0011	0.0064	0.0012	0.0074
1	R1	0.7920	0.7920	0.9503	1.0000
2	B2	0.0865	0.3460	0.0788	0.3154
2	DE	0.6454	1.0000	0.9152	1.0000
2	H-	0.0232	0.1158	0.0382	0.1912
2	My	0.8983	1.0000	0.9814	1.0000
2	NE	<.0001	<.0001	<.0001	<.0001
2	R1	0.6666	1.0000	0.8267	1.0000
3	B2	0.0908	0.1846	0.3635	0.5662
3	DE	0.0049	0.0245	0.0253	0.1263
3	H-	0.0342	0.1367	0.1576	0.4728
3	My	0.0615	0.1846	0.2831	0.5662
3	NE	0.0651	0.1846	0.0724	0.2897
3	R1	0.0024	0.0147	0.0043	0.0259
4	B2	0.0087	0.0330	0.1778	0.5334
4	DE	0.4473	0.4473	0.0234	0.1170
4	H-	0.0082	0.0330	0.9269	0.9269
4	My	0.0039	0.0195	0.2036	0.5334
4	NE	0.0012	0.0074	0.0044	0.0264
4	R1	0.0083	0.0330	0.0381	0.1523

References

1. Shao, C. *et al.* Analysis-preserving video microscopy compression via correlation and mathematical morphology. *Microsc. Res. Tech.* **00,** n/a–n/a (2015).

2. *Handbook Of Biological Confocal Microscopy*. (Springer US, 2006). doi:10.1007/978-0-387-45524-2

3. Stigler, S. M. Francis Galton ' s Account of the Invention of Correlation. *Stat. Sci.* **4,** 73–79 (1989).

4. van Elteren, P. H. On the combination of independent two sample tests of Wilcoxon. *Bull. Inst. Int. Stat.* 351–361 (1960).

5. Holm, S. A simple sequentially rejective multiple test procedure. *Scand. J. Stat.* 65–70 (1979).

6. Box, G. E. P. & Cox, D. R. An analysis of transformations. *J. R. Stat. Soc. Ser. B* 211–252 (1964).

7. Lin, D. C., Dimitriadis, E. K. & Horkay, F. Robust strategies for automated AFM force curve analysis--I. Non-adhesive indentation of soft, inhomogeneous materials. *J Biomech Eng* **129,** 430–440 (2007).

8. Thomas, G., Burnham, N. a, Camesano, T. A. & Wen, Q. Measuring the mechanical properties of living cells using atomic force microscopy. *J. Vis. Exp.* 1–8 (2013). doi:10.3791/50497

9. Vargas-Pinto, R., Gong, H., Vahabikashi, a & Johnson, M. The effect of the endothelial cell cortex on atomic force microscopy measurements. *Biophys. J.* **105,** 300–9 (2013).
